# The effectiveness of non-invasive brain stimulation on arousal and alertness in patients in coma or persistent vegetative state after traumatic brain injury: Protocol of systematic review and network meta-analysis

**DOI:** 10.1097/MD.0000000000012321

**Published:** 2018-09-14

**Authors:** Yabin Li, Xianggui Luo, Miao Wan, Jiao Li, Hongxia Wang, Dang Wei, Haixia Feng

**Affiliations:** a Department of Neurological Rehabilitation, Rehabilitation Central Hospital of Gansu Province; b The First Clinical Medical College of Lanzhou University, Lanzhou, China; c Department of Public Health Sciences, Karolinska Institute, Stockholm, Sweden.

**Keywords:** coma state, network meta-analysis, non-invasive brain stimulation, persistent vegetative state, systematic review, traumatic brain injury

## Abstract

**Background:**

Traumatic brain injury is a leading cause of death and disability worldwide. The survivors usually suffer from disorders of consciousness, especially coma state and persistent vegetative state. For these patients, there is no standard treatment for them, but non-invasive brain stimulations are considered as relatively more acceptable treatments. However, the knowledge regarding the relative effectiveness and the rank of the effectiveness of the non-invasive brain stimulations is limited. Thus, in this study, we aim to conduct a systematic review and network meta-analysis to evaluate the effect of non-invasive train stimulations on arousal and alertness in patients in a coma or persistent vegetative state after traumatic brain injury.

**Methods and analysis:**

A comprehensive search strategy will be performed in the relevant databases (MEDLINE, EMBASE, Cochrane Central Register of Controlled Trials, World Health Organization (WHO) International Clinical Trials Registry Platform (ICTRP), Chinese Biomedical Literature Database, China National Knowledge Infrastructure, Wan Fang Data). The random or quasi-random controlled trails focusing on the effectiveness of the non-invasive brain stimulations will be included. The risk of bias for the included studies will be appraised using the Cochrane collaboration tool for assessing risk of bias. The standard pairwise meta-analysis and a Bayesian network meta-analysis will be conducted.

**Ethics and dissemination:**

This research is a systematic review and network meta-analysis. Thus, there is no requirement of ethical approval and patient informed consent.

**PROSPERO registration number:**

CRD42018104945.

## Abstract 2

Supplemental Digital Content is available in the text

## 1 Background

Traumatic brain injury (TBI) is a leading cause of death and disability worldwide, particularly in Southeast Asian and Western Pacific regions.^[1]^ In each year, about 5.48 million people (73 cases per 100,000) suffer from severe TBI,^[1]^ of which most survivors get disorders of consciousness (DOC). Majority of these patients turn into a coma state, and generally the coma survivors may enter a gradual recovery process of consciousness^[2]^—firstly into unresponsive wakefulness syndrome (UWS) which is also called persistent vegetative state (PVS) if the patient has kept in a vegetative state for more than 4 weeks, then into a minimally conscious state (MCS) until getting out from DOC. But around 10%^[3,4]^ to 30%^[5]^ coma survivors remain in PVS even after about 1 month. Moreover, about 50% of patients who have been experienced PVS for at least 4 weeks after head injury remain in this state 1 year post-TBI ^[3,4,6,7]^; and approximately 23% of MCS patients don’t recover their consciousness 1 year after TBI.^[8,9]^

From the financial perspective, it brings great burden to the family and society. For example, a previous review showed that the costs per case were from about $34,000 for mild to about $60,000 for moderate TBI.^[10]^ Unfortunately, so much expense is spent, but there is no standard care which may result in cost-effectiveness for these patients.^[11]^ Even though several previous systematic reviews^[12–16]^ have systematically evaluated the potential effective treatments for these patients, the credible evidence has not been found because of a small number of relevant trails, the small sample size, inconsistency, old evidence grading system, and so on. Owing to the limited evidence on the effect of the interventions, the non-invasive brain stimulations (including right median nerve stimulation [RMNS], sensory stimulation [SS], transcranial magnetic stimulation [TMS], transcranial direct current stimulation [TDCS], and virtual reality [VR]), with little ham and adverse effect, are considered as relatively more acceptable treatments. In the recent years, more new relevant studies assessing the effectiveness of non-invasive brain stimulations have been published or are conducting.^[17–22]^ With the newly published studies and more patients participating in the researches, we are likely able to confirm the effectiveness of non-invasive brain stimulations. In addition, the existing systematic reviews only conducted the pairwise meta-analysis to compare the efficacy of different types of non-invasive brain stimulations by head-to-head. The knowledge regarding the rank of the effectiveness of the non-invasive brain stimulations is limited. To the best of our knowledge, network meta-analysis (NMA)^[23]^ is developed to address this problem, which is able to confirm the relative effectiveness among all the potential interventions and rank the order of the effect of interventions when head-to-head comparisons lack. Therefore, in this study, we aim to conduct a comprehensive systematic review and network meta-analysis to evaluate the effectiveness of the non-invasive brain stimulations on arousal and alertness in patients in coma or persistent vegetative state after traumatic brain injury.

## 2 Methods

We have registered the protocol of this systematic review and network meta-analysis on the PROSPERO international prospective register of systematic reviews (Register number: CRD42018104945). The reporting of this protocol was in accordant with the preferred reporting items for systematic review and meta-analysis protocol (PRISMA-P),^[24,25]^ and the PRISMA extension statement for reporting of systematic reviews incorporating network meta-analyses of healthcare interventions.^[26]^

### 2.1 Eligibility criteria

We plan to include the studies which meet the following criteria: the patients in the coma or vegetative state after traumatic brain injury without limitation on the age; aim to evaluate the effectiveness of the non-invasive brain stimulations through the comparisons of: one of the non-invasive stimulations (including SS, TDCS, TMS, RMNS, and VR) versus each other or usual care; multi-stimulations versus uni-stimulation or usual care; different types of combination of multi-stimulations; report at least one of the following outcomes: the rate of wakening, duration of unconsciousness, level of consciousness measured by the Glasgow Coma Scale (GCS), level of cognitive functioning (LCF), functional outcomes measured by Glasgow Outcome Scale (GOS) or Disability Rating Scale (DRS), adverse effects (ie, increased intracranial pressure); random or quasi-random controlled trials (during the study screening stage, the relevant systematic reviews, and meta-analyses will also be included for tracking their references).

### 2.2 Information source

A comprehensive search strategy will be developed, which will be mainly led by an author (DW) together with the group discussion. We will search the databases below: Cochrane Central Register of Controlled Trials, EMBASE, MEDLINE (via PubMed), Chinese Biomedical Literature Database, China National Knowledge Infrastructure, and Wan Fang Data. The ongoing studies and the studies which have been completed but not yet published will be searched through the World Health Organization (WHO) International Clinical Trials Registry Platform (ICTRP) (http://apps.who.int/trialsearch/Default.aspx).

We will check the references of included articles to retrieve further relevant studies.

### 2.3 Search strategy

The electronic search will be performed from the inception of databases to August 2018 and without any language limitation. We plan to use the search terms regarding the targeted participants together with the non-invasive sensory stimulations, which the details of the search strategy in PubMed can be seen below:

1. “Coma, Post-Head Injury” [Mesh] OR “Persistent Vegetative State” [Mesh] OR “Brain Injuries/complications” [Mesh] OR “vegetative state” [Title/Abstract] OR coma [Title/Abstract]
2. “Electric Stimulation” [Mesh] OR “Transcranial Direct Current Stimulation” [Mesh] OR “Transcranial Magnetic Stimulation” [Mesh] OR “Virtual Reality Exposure Therapy” [Mesh] OR “sensory stimulation” [Title/Abstract] OR “transcranial direct current stimulation” [Title/Abstract] OR “transcranial magnetic stimulation” [Title/Abstract] OR “right median nerve stimulation” [Title/Abstract] OR “virtual reality”[Title/Abstract] OR videogame [Title/Abstract] OR tdcs [Title/Abstract] OR tms [Title/Abstract] OR rmns [Title/Abstract] OR vr [Title/Abstract]
3. 1 AND 2

More information on search strategy in the rest databases can be found in the supplement file.

### 2.4 Study selection and data extraction

Firstly, the records retrieved from the databases will be imported to EndNote X7 literature management software by DW who is professional on searching the electronic databases and conducting a systematic review. Then, pairs of researchers will screen the records independently by reviewing the title and abstract. In the next step, the pairs of researchers will review the full-texts of the potential eligible studies independently.

With an electronic form, the following data will be extracted by pairs of researchers independently: study characteristics (such as title, first author, publication type, publication year, country, journal, the sponsor), study design (inclusion and exclusion criteria, generation of allocation sequence, allocation concealment and blinding, length of follow-up), participant data (sample size, race, age, diagnosis [condition] of the patients, diagnostic criteria, co-morbidities, lost/withdrawal), details of interventions of interest (type, protocol, frequency, duration), and outcomes (measured tools, time point, results).

Before study selection and data extraction, we plan to do a pilot test for each assignment respectively in order to ensure high inter-rater reliability among the researchers. When there are disagreements between a pair of researchers, discussion will be organized and the conflicts will be solved by a third researcher (DW).

### 2.5 Risk of bias assessment

The tool for assessing risk of bias developed by Cochrane collaboration^[27]^ will be applied to appraise the risk of bias of included randomized controlled trails (RCTs) by pairs of independent researchers. There are 7 domains in this tool, including sequence generation, allocation concealment, blinding of participants and personnel, blinding of outcome assessment, incomplete outcome data, selective outcome reporting, and other sources of bias. Each domain is evaluated as low, high, unclear risk of bias. The support for the adjustments will be recorded. Discussion will be organized and conflict will be solved by DW, when where are disagreements.

### 2.6 Data synthesis

#### 2.6.1 Dealing with missing data

For the important data such as the standard deviations or standard errors of the continue outcomes, if they were not reported in the articles, we will firstly calculate it by ourselves based on the reported information such as confidence interval (CI) or *P*-value. If the reported data is not enough for calculation, we plan to the authors to obtain the data. What's worse, if no data is not successfully got from the authors, the methods suggested by Furukawa et al^[28]^ will be used to retrieve the missing data. The assumptions derived from these data will be tested through sensitivity analysis.

#### 2.6.2 Standard pairwise meta-analysis

We will pool odd rations (ORs) and mean differences (MDs) with 95% CI for the dichotomous and continue outcomes respectively by performing the pairwise meta-analysis in STATA V.12.0 (Stata Corporation, CollegeStation, Texas). The effects model will be chose after assessing the heterogeneity by *I*^*2*^ statistics. If *I*^*2*^ ≤50%, we will use the Mantel–Haenszel fixed effects model to pool the data. Otherwise, firstly subgroup analysis and meta-regression will be performed to test the sources of heterogeneity. If no evidence on clinical heterogeneity is found, the Mantel–Haenszel random effects model will be chose. But if there is apparent clinical heterogeneity, we plan to do subgroup analysis (if available) or only describe the results of the included studies respectively. With regard to the assessment of reporting bias, the Begg and Egger funnel plot method will be used.^[29,30]^ And the contour-enhanced funnel plot will be used to help to distinguish asymmetry, if multi-factors lead to publication bias.^[31]^

#### 2.6.3 Network meta-analysis

Firstly, a network plot of the treatment network of comparisons across trials will be drawn to assess whether the network meta-analysis is available. In the network geometry, nodes and edges stand for the interventions and the head-to-head comparisons between them separately. The bigger the node is, the larger the sample size focused on the intervention is; and the thicker the edge is, the more trails focused on the comparison there are.

Next, we plan to conduct the Bayesian network meta-analysis by the code from Dias et al^[32]^ in WinBUGS 1.4.3. The pooled estimation and the rank of the effect of the interventions will be obtained according to the Markov Chains Monte Carlo method. We plan to run three Markov Chains simultaneously and firstly generate 50,000 simulations for each chain which these simulations will be discarded as the ‘burn-in’ period; 100,000 subsequent simulations will be chose for the posterior summaries. We will evaluate the model convergence by trace plots and Brooks–Gelman-Rubin plots,^[33]^ and assess the statistical heterogeneity in the entire network according to the magnitude of heterogeneity variance parameter (*I*^*2*^ or τ^2^) estimated from the network meta-analysis models using R-3.2.2 software. If a loop connecting three arms exists, inconsistency between direct and indirect comparisons will be evaluated using a node splitting method.^[34]^ The choices between fixed and random effect models, consistent and inconsistent models, will be made by comparing the deviance information criteria (DIC) for each model.^[32,35]^ The model with the lowest DIC will be preferred (differences >3 are considered significant).

With regard to the sources of heterogeneity or inconsistency in the entire network, the network meta-regression or subgroup analysis will be conducted. We will conduct the network meta-regression under the random effects models to assess the potential effect moderators such as follow-up and sample size.

If there are enough trials for comparison, we plan to conduct a sensitivity analysis by excluding trials which miss the relative data and the trials with a total sample size of <50 randomized patients.

### 2.7 Quality of evidence

We will use the Grading of Recommendation, Assessment, Development and Evaluation approach (GRADE) to assess the quality of evidence—how much confidence we have on the effect estimation.^[36]^ The process will be performed on the platform of GRADEpro – GDT (https://gradepro.org/).

### 2.8 Ethics and dissemination

This research is a systematic review and network meta-analysis. Thus, there is no requirement of ethical approval and patient informed consent.

## 3 Discussion

To our knowledge, this is the first network meta-analysis which compares the effectiveness of different non-invasive brain stimulations on arousal and alertness in patients in coma or persistent vegetative state after traumatic brain injury. The quality of evidence rated through GRADE approach in this review would clearly inform the evidence users the extent to which we believe that the findings are truth. Thus, the findings of this review would be likely to inform the decision making on healthcare for those patients in clinical practice.

## Author contributions

Contributors: Conception and design of this systematic review and Bayesian network meta-analysis (Yabin Li, Xianggui Luo, Miao Wan, Dang Wei, Haixia Feng); tested the feasibility of the study (Yabin Li, Xianggui Luo, Miao Wan, Jiao Li, Hongxia Wang); developed the search strategy (Dang Wei, Miao Wan, Xianggui Luo); drafted this protocol (Yabin Li, Xianggui Luo, Miao Wan, Haixia Feng), revised the protocol (Yabin Li, Xianggui Luo, Miao Wan, Jiao Li, Hongxia Wang, Dang Wei). All authors provided critical revisions of the protocol and approved the final article.

**Conceptualization:** Yabin Li, Xianggui Luo, Miao Wan, Dang Wei, Haixia Feng.

**Investigation:** Jiao Li, Hongxia Wang.

**Methodology:** Xianggui Luo, Miao Wan, Dang Wei.

**Supervision:** Haixia Feng.

**Writing – original draft:** Yabin Li, Xianggui Luo, Miao Wan, Haixia Feng.

**Writing – review & editing:** Yabin Li, Xianggui Luo, Miao Wan, Jiao Li, Hongxia Wang, Dang Wei.

## Supplementary Material

Supplemental Digital Content

## References

[1] Dewan MC Rattani A Gupta S Estimating the global incidence of traumatic brain injury. J Neurosurg 2018;1–8. 10.3171/2017.10.JNS17352 29701556

[2] Jennett B Teasdale G Braakman R Prognosis of patients with severe head injury. Neurosurgery 1979;4:283–9. 450225 10.1227/00006123-197904000-00001

[3] Braakman R Jennett WB Minderhoud JM Prognosis of the posttraumatic vegetative state. Acta Neurochir (Wien) 1988;95:49–52. 3218553 10.1007/BF01793082

[4] Levin HS Saydjari C Eisenberg HM Vegetative state after closed-head injury. A Traumatic Coma Data Bank Report. Arch Neurol 1991;48:580–5. 2039378 10.1001/archneur.1991.00530180032013

[5] Choi SC Barnes TY Bullock R Temporal profile of outcomes in severe head injury. J Neurosurg 1994;81:169–73. 8027796 10.3171/jns.1994.81.2.0169

[6] Medical aspects of the persistent vegetative state (2). The Multi-Society Task Force on PVS. N Engl J Med 1994;330:1572–9. 8177248 10.1056/NEJM199406023302206

[7] Sazbon L Groswasser Z Time-related sequelae of TBI in patients with prolonged post-comatose unawareness (PC-U) state. Brain Inj 1991;5:3–8. 2043905 10.3109/02699059108998505

[8] Katz DI Polyak M Coughlan D Natural history of recovery from brain injury after prolonged disorders of consciousness: outcome of patients admitted to inpatient rehabilitation with 1-4 year follow-up. Prog Brain Res V 177 2009;73–88. 10.1016/S0079-6123(09)17707-5 19818896

[9] Lammi MH Smith VH Tate RL The minimally conscious state and recovery potential: a follow-up study 2 to 5 years after traumatic brain injury. Arch Phys Med Rehabil 2005;86:746–54. 15827927 10.1016/j.apmr.2004.11.004

[10] Runge JW The cost of injury. Emerg Med Clin North Am 1993;11:241–53. 8432252

[11] Cossu G Therapeutic options to enhance coma arousal after traumatic brain injury: State of the art of current treatments to improve coma recovery. Br J Neurosurg 2014;28:187–98. 24090192 10.3109/02688697.2013.841845

[12] Vanhoecke J Hariz M Deep brain stimulation for disorders of consciousness: systematic review of cases and ethics. Brain Stimul 2017;10:1013–23. 28966051 10.1016/j.brs.2017.08.006

[13] Lombardi F Taricco M De Tanti A Sensory stimulation for brain injured individuals in coma or vegetative state. Cochrane Database Syst Rev 2002;2:CD001427. 10.1002/14651858.CD001427 PMC7045727 12076410

[14] Padilla R Domina A Effectiveness of sensory stimulation to improve arousal and alertness of people in a coma or persistent vegetative state after traumatic brain injury: a systematic review. Am J Occup Ther 2016;70: 7003180030p1-8. 10.5014/ajot.2016.021022 27089287

[15] Cicerone KD Participation after multidisciplinary rehabilitation for moderate to severe traumatic brain injury in adults: a systematic review. Arch Phys Med Rehabil 2013;94:1421–3. 23800407 10.1016/j.apmr.2013.04.003

[16] Pietrzak E Pullman S1 McGuire A Using virtual reality and videogames for traumatic brain injury rehabilitation: a structured literature review. Games Health J 2014;3:202–14. 26192369 10.1089/g4h.2014.0013

[17] Leśniak M Polanowska K Seniów J Effects of repeated anodal tDCS coupled with cognitive training for patients with severe traumatic brain injury: a pilot randomized controlled trial. J Head Trauma Rehabil 2014;29:E20–9. 23756431 10.1097/HTR.0b013e318292a4c2

[18] Lei J Wang L Gao G Right median nerve electrical stimulation for acute traumatic coma patients. J Neurotrauma 2015;32:1584–9. 25664378 10.1089/neu.2014.3768

[19] Angelakis E Liouta E Andreadis N Transcranial direct current stimulation effects in disorders of consciousness. Arch Phys Med Rehabil 2014;95:283–9. 24035769 10.1016/j.apmr.2013.09.002

[20] Martino Cinnera A Bonnì S Iosa M Clinical effects of non-invasive cerebellar magnetic stimulation treatment combined with neuromotor rehabilitation in traumatic brain injury. A single case study. Funct Neurol 2016;31:117–20. 27358225

[21] Conde V Andreasen SH Petersen TH Alterations in the brain's connectome during recovery from severe traumatic brain injury: protocol for a longitudinal prospective study. BMJ Open 2017;7:e016286. 10.1136/bmjopen-2017-016286 PMC5541610 28615277

[22] Neville IS Hayashi CY El Hajj SA Repetitive Transcranial Magnetic Stimulation (rTMS) for the cognitive rehabilitation of traumatic brain injury (TBI) victims: study protocol for a randomized controlled trial. Trials 2015;16:440. 26438108 10.1186/s13063-015-0944-2 PMC4594992

[23] Mills EJ Thorlund K Ioannidis JP Demystifying trial networks and network meta-analysis. BMJ 2013;346:f2914. 23674332 10.1136/bmj.f2914

[24] Moher D Shamseer L Clarke M Preferred reporting items for systematic review and meta-analysis protocols (PRISMA-P) 2015 statement. Syst Rev 2015;4:1. 25554246 10.1186/2046-4053-4-1 PMC4320440

[25] Shamseer L Moher D Clarke M Preferred reporting items for systematic review and meta-analysis protocols (PRISMA-P) 2015: elaboration and explanation. BMJ 2015;350:g7647. 25555855 10.1136/bmj.g7647

[26] Hutton B Salanti G Caldwell DM The PRISMA extension statement for reporting of systematic reviews incorporating network meta-analyses of health care interventions: checklist and explanations. Ann Intern Med 2015;162:777–84. 26030634 10.7326/M14-2385

[27] Cochrane Collaboration. Cochrane handbook for systematic reviews of interventions version 5.1.0. [www.cochrane-handbook.org].

[28] Furukawa TA Barbui C Cipriani A Imputing missing standard deviations in meta-analyses can provide accurate results. J Clin Epidemiol 2006;59:7–10. 16360555 10.1016/j.jclinepi.2005.06.006

[29] Egger M Davey Smith G Schneider M Bias in meta-analysis detected by a simple, graphical test. BMJ 1997;315:629–34. 9310563 10.1136/bmj.315.7109.629 PMC2127453

[30] Begg CB Mazumdar M Operating characteristics of a rank correlation test for publication bias. Biometrics 1994;50:1088–101. 7786990

[31] Peters JL Sutton AJ Jones DR Contour-enhanced meta-analysis funnel plots help distinguish publication bias from other causes of asymmetry. J Clin Epidemiol 2008;61:991–6. 18538991 10.1016/j.jclinepi.2007.11.010

[32] Dias S Sutton AJ Ades AE Evidence synthesis for decision making 2: a generalized linear modeling framework for pairwise and network meta-analysis of randomized controlled trials. Med Decis Making 2013;33:607–17. 23104435 10.1177/0272989X12458724 PMC3704203

[33] Gelman A Rubin DB Inference from iterative simulation using multiple sequences. Statist Sci 1992;7:457–72.

[34] Lu G Ades AE Combination of direct and indirect evidence in mixed treatment comparisons. Stat Med 2004;23:3105–24. 15449338 10.1002/sim.1875

[35] David JS Nicola GB Bradley PC Bayesian measures of model complexity and fit. J R Statist Soc B 2002;64:583–639.

[36] Puhan MA Schünemann HJ Murad MH A GRADE Working Group approach for rating the quality of treatment effect estimates from network meta-analysis. BMJ 2014;349:g5630. 25252733 10.1136/bmj.g5630

