# Supplementary material for: The effectiveness of non-invasive brain stimulation on arousal and alertness in patients in coma or persistent vegetative state after traumatic brain injury: Protocol of systematic review and network meta-analysis
Source: Medicine (Baltimore). 2018 Sep 14;97(37):e12321. doi: 10.1097/MD.0000000000012321 (PMC6155968; doi:10.1097/MD.0000000000012321)
Supplement: Supplemental Digital Content [file medi-97-e12321-s001.doc]

**Search strategy**

**1. PubMed**

#1 "Coma, Post-Head Injury"[Mesh] OR "Persistent Vegetative State"[Mesh] OR "Brain Injuries/complications"[Mesh] OR "vegetative state"[Title/Abstract] OR coma[Title/Abstract]

#2 "Electric Stimulation"[Mesh] OR "Transcranial Direct Current Stimulation"[Mesh] OR "Transcranial Magnetic Stimulation"[Mesh] OR "Virtual Reality Exposure Therapy"[Mesh] OR "sensory stimulation"[Title/Abstract] OR "transcranial direct current stimulation"[Title/Abstract] OR "transcranial magnetic stimulation"[Title/Abstract] OR "right median nerve stimulation"[Title/Abstract] OR "virtual reality"[Title/Abstract] OR videogame[Title/Abstract] OR tdcs[Title/Abstract] OR tms[Title/Abstract] OR rmns[Title/Abstract] OR vr[Title/Abstract]

#3 #1 AND #2

**2. Embase**

#1 'persistent vegetative state'/exp OR 'traumatic brain injury'/exp OR 'vegetative state':ab,ti OR coma:ab,ti

#2 'transcutaneous electrical nerve stimulation'/exp OR 'transcranial direct current stimulation'/exp OR 'transcranial magnetic stimulation'/exp OR 'virtual reality exposure therapy'/exp OR ‘sensory stimulation’:ab,ti OR ‘transcranial direct current stimulation’:ab,ti OR ‘transcranial magnetic stimulation’:ab,ti OR ‘right median nerve stimulation’:ab,ti OR ‘virtual reality’:ab,ti OR videogame:ab,ti OR tdcs:ab,ti OR tms:ab,ti OR rmns:ab,ti OR vr:ab,ti

#3 #1 AND #2

#4 #3 AND [embase]/lim NOT ([embase]/lim AND [medline]/lim)

**3. Cochrane Central Register of Controlled Trials**

#1 MeSH descriptor: [Persistent Vegetative State] explode all trees OR MeSH descriptor: [Coma, Post-Head Injury] explode all trees OR "vegetative state":ti,ab,kw OR coma:ti,ab,kw

#2 MeSH descriptor: [Transcutaneous Electric Nerve Stimulation] explode all trees OR MeSH descriptor: [Transcranial Direct Current Stimulation] explode all trees OR MeSH descriptor: [Transcranial Magnetic Stimulation] explode all trees OR MeSH descriptor: [Virtual Reality] explode all trees OR “sensory stimulation”:ti,ab,kw OR “transcranial direct current stimulation”:ti,ab,kw OR “transcranial magnetic stimulation”:ti,ab,kw OR “right median nerve stimulation”:ti,ab,kw OR “virtual reality”:ti,ab,kw OR videogame:ti,ab,kw OR tdcs:ti,ab,kw OR tms:ti,ab,kw OR rmns:ti,ab,kw OR vr:ti,ab,kw

#3 #1 AND #2

**4. WHO ICTRP search portal**

#1 “sensory stimulation” OR “Transcranial Direct Current Stimulation” “Transcranial Magnetic Stimulation” OR “virtual reality” OR videogame: in titile

**5. Chinese Biomedical Literature Database**

#1 “昏迷”[不加权:扩展] OR “植物状态”[不加权:扩展] OR “重度颅脑损伤”[不加权:扩展] OR “昏迷”[常用字段:智能] OR “植物状态”[常用字段:智能] OR “植物人”[常用字段:智能] OR “颅脑损伤”[常用字段:智能]

#2 “经颅直流电刺激” [常用字段:智能] OR “经颅电刺激”[常用字段:智能] OR “经颅磁刺激” [常用字段:智能] OR “正中神经电刺激” [常用字段:智能] OR “VR眼镜”[常用字段:智能] OR “镜像神经元”[常用字段:智能]

#3 #1 AND #2

**6. China National Knowledge Infrastructure**

#1 SU=(‘昏迷’+’植物状态’+’植物人’+’颅脑损伤’)

#2 SU=(‘经颅直流电刺激’+’经颅电刺激’+’经颅磁刺激’ +’正中神经电刺激’+’VR眼镜’+’镜像神经元’)

#3 #1 AND #2

**7. Wan Fang Data**

#1 主题:(昏迷 OR植物状态 OR 植物人 OR 颅脑损伤)

#2 主题:(经颅直流电刺激 OR 经颅电刺激 OR 经颅磁刺激 OR 正中神经电刺激OR VR眼镜 OR 镜像神经元)

#3 #1 AND #2
